# Supplementary material for: Investigation of the Effect of Rice Bran Content on the Antioxidant Capacity and Related Molecular Conformations of Plant-Based Simulated Meat Based on Raman Spectroscopy
Source: Foods. 2022 Nov 6;11(21):3529. doi: 10.3390/foods11213529 (PMC9657750; doi:10.3390/foods11213529)
Supplement: Supplementary file 1 [file foods-11-03529-s001.zip › foods-1907833-supplementary.pdf]

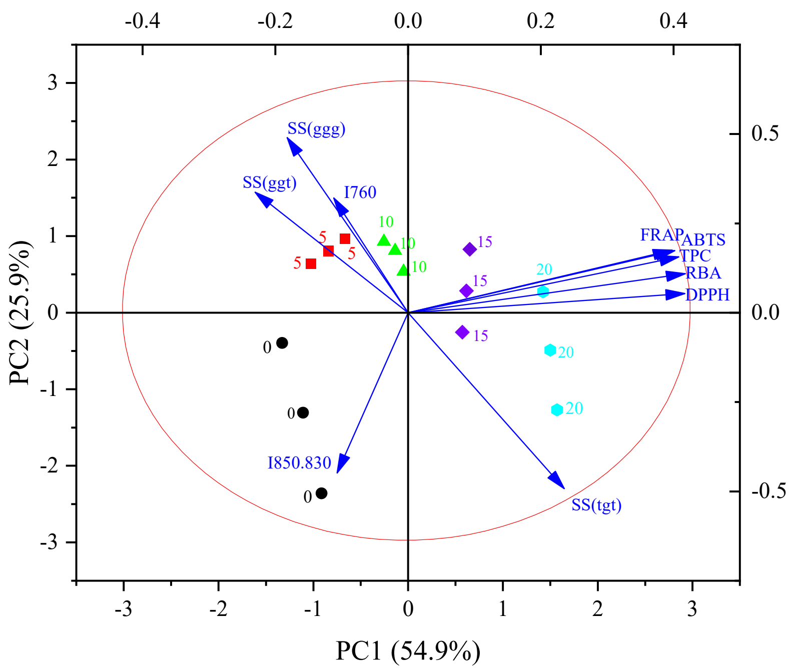

Fig. S1. Principal component analysis of RB-SPI plant simulated meat

Noted: RBA -- RB addition; TPC -- total phenol content; DPPH -- DPPH radical scavenging method; ABTS -- ABTS radical scavenging method; FRAP -- FRAP antioxidant capacity; I760 -- intensity of the tryptophan band at 760 cm^{-1}; I860.830 -- tyrosine doublet; SS(ggg) -- amount of disulphide bridges in the g-g-g conformation; SS(ggt) -- amount of disulphide bridges in the g-g-t conformation; SS (tgt) -- amount of disulphide bridges in the t-g-t conformation; black circles for 0% RB; red squares for 5% RB; green triangles for 10% RB; purple diamonds for 15% RB; blue hexagons for 20% RB.
